# *ENO2* Affects the Seed Size and Weight by Adjusting Cytokinin Content and Forming ENO2-bZIP75 Complex in *Arabidopsis thaliana*

**DOI:** 10.3389/fpls.2020.574316

**Published:** 2020-08-26

**Authors:** Zijin Liu, Lamei Zheng, Li Pu, Xiaofeng Ma, Xing Wang, Yu Wu, Hainan Ming, Qing Wang, Genfa Zhang

**Affiliations:** ^1^Beijing Key Laboratory of Gene Resource and Molecular Development, College of Life Sciences, Beijing Normal University, Beijing, China; ^2^Biotechnology Research Institute, Chinese Academy of Agriculture Sciences, Beijing, China; ^3^Institute of Radiation Botany, Beijing Radiation Center, Beijing, China

**Keywords:** *ENO2*, seed size, seed weight, *Arabidopsis*, bZIP75, plant hormone

## Abstract

*Arabidopsis thaliana ENO2* (*AtENO2*) encodes two proteins AtENO2 (enolase) and AtMBP-1 (c-Myc binding protein 1-like). The loss of *AtENO2* function causes the constitutive developmental defects which are correlated with reduced enolase activity, but not AtMBP-1 transcript abundance. However, the regulation mechanism of *AtENO2* on the seed properties is still not clear. In this study, we found that the mutation of *AtENO2* reduced the seed size and weight. The level of glucose in seed was significantly elevated but that of starch was decreased in *AtENO2* mutants compared to WT plants. We also found that *AtENO2* mutation reduced the content of cytokinin which resulted in smaller cotyledons. The RNA-seq data showed that there were 1892 differentially expressed genes and secondary metabolic pathways were significantly enriched. Instead of AtMBP-1, AtENO2 protein interacted with AtbZIP75 which may mediate the secondary metabolism. Therefore, *ENO2* alters the size and weight of seeds which is not only regulated by the content of cytokinin and secondary metabolism, but may be affected by the interaction of ENO2 and bZIP57. These results are helpful to understand the novel function of *AtENO2* which provide a foundation for further exploration of the key candidate genes for crop breeding.

## Introduction

In the field of agricultural production, seed size and weight play important roles in the plant fitness and crop yield (Moles et al., 2005; Liu et al., 2016). The size of seeds negatively affects the seed numbers and the larger seeds positively develop into larger seedling, which are much stronger to endure abiotic stresses (Coomes and Grubb, 2003; Li et al., 2013). In angiosperms, the development of seeds originates the fusion of one sperm and one egg cell or two polar nuclei, which produces a diploid embryo and a triploid endosperm (Chaudhury et al., 2001). In the mature seeds of *Arabidopsis*, only one layer endosperm surrounds the embryo and the endosperm is enveloped by seed coat which is formed by several layers of specialized maternal cell types that impart protection, improve dormancy, and germination (Haughn and Chaudhury, 2005). Therefore, as the three major components in *Arabidopsis thaliana*, the coordinated growth and development of seed coat, endosperm, and embryo will determine the seed size and weight.

The regulatory network of genes that involved in the growth and development of maternal and zygotic tissues is crucial for seed size/weight. Recently, extensive studies have been made in establishing what metabolism molecule integrates plant responses to environmental stresses into the control of seed size and weight (Meng et al., 2018). It has been reported that soluble sugar accumulation can influence seed size *via AN3-YDA* (*ANGUSTIFOLIA3-YODA*) gene cascade and integration of environmental and development (or metabolic), and control seed weight by sugar and ethylene metabolisms in *Arabidopsis* (Meng et al., 2017; Meng et al., 2018). The signaling pathways that may affect seed size and weight have been identified, including the ubiquitin-proteasome pathway, G-protein signaling, mitogen-activated protein kinase signaling, transcriptional regulators, HAIKU (IKU) pathway, and phytohormones (Li and Li, 2016; Li et al., 2019).

The *ENO* gene are widely distributed and highly conserved in vertebrates and plants which encodes a glycolytic metalloenzyme enolase (ENO2) that catalyzes the dehydration of 2-phospho-d-glycerate (2-PGA) to phosphoenolpyruvate (PEP) (Liu Z. J. et al., 2019). In *Arabidopsis*, there are totally three *ENO* genes, *At1g74030* (*ENO1*), *At2g36530* (*ENO2*, also referred to *LOS2*, Lee et al., 2002) and *At2g29560* (*ENO3*). Subcellular localization of enolase isoforms displayed that ENO1 and ENO3 are located in chloroplast and cytoplasm, respectively. However, ENO2 was observed in cytosol and nuclei (Andriotis et al., 2010). In addition, ENO1 and ENO2 proteins possess enolase activity but ENO3 does not (Lee et al., 2002; Prabhakar et al., 2009; Andriotis et al., 2010). *ENO2* is expressed in all organs and developmental stages, and more highly in root and silique (Andriotis et al., 2010). *Arabidopsis ENO2* (*AtENO2*) is a multifunctional gene encoding two proteins AtENO2 and AtMBP-1 (c-Myc binding protein 1-like). AtENO2 is 444 amino acids in length, while AtMBP-1 is 352 amino acids in length which lacks enzymatic activity and feedback-repressed the enolase activity (Eremina et al., 2015; Liu et al., 2018). The absence of AtENO2 (enolase) causes the defective growth and reproduction, such as smaller plant, reduced germination rate of pollen, impaired floral organogenesis, and impaired male gametophyte (Eremina et al., 2015). The overexpression of AtMBP-1 (in *35S:AtMBP-1-YFP* and *35S:LOS2-YFP* lines) produced the phenotypes that strongly resemble those of the *los2/eno2* knock-out lines (Kang et al., 2013). Therefore, *AtENO2* plays a pleiotropic role in regulating growth, development, and fertility of plant organs. However, it is still not clear that the effect of *AtENO2* on the seed properties and what the regulation mechanism is. Eremina et al. (2015) pointed that *ENO2* mutants and AtMBP-1 overexpressing plants show a strong phenotypic resemblance with other mutants impaired in glycolysis, and the constitutive developmental defects in plant are correlated with a strong reduction in enolase activity, but not with a reduction in AtMBP-1 transcript abundance. Our previous study also demonstrated that AtMBP-1 is predominant in tolerance of adult Arabidopsis to abiotic stresses (Liu Z. J. et al., 2019).

In this work, we found that mutation of *AtENO2* reduced the seed size and weight due to decreasing the content of cytokinin. The data of carbohydrate detection and RNA-seq showed that the metabolism and especially the secondary metabolism were disturbed in the *AtENO2* T-DNA insertion mutant. In addition, we found that AtENO2 interacted with *Arabidopsis* basic-leucine zipper 75 (AtbZIP75) instead of AtMBP-1. Thus, AtbZIP75 may participate in the process of seed development. Our study showed novel insights into *AtENO2* function in the growth and development of seed, and provide a promising target gene for genetic manipulation of crop breeding.

## Materials and Methods

### Plant Material and Growth Conditions

The wild type (WT) is *Arabidopsis* Columbia (Col-0), and the *AtENO2/AtLOS2* T-DNA insertion mutants (*los2-2*, SALK_021737; *los2-3*, SALK_077784; *los2-4*, SAIL_208_B09) in the wild type background were obtained from Arabidopsis Biological Resource Center (ABRC, Columbus, Ohio). The details of mutation have been descripted by Eremina et al. (2015). Because *los2-3* and *los2-4* suffer from embryo lethality and produce no seeds (Eremina et al., 2015), we used the mutant *los2-2* (called *eno2*^−^ in our study) for subsequent research. The *eno2*^−^/*35S:AtENO2* and WT/*35S:AtENO2* lines were obtained from T_0_ to T_3_ by screening on the MS medium supplemented with 50 μg/mL of hygromycin. The seeds used in the research were soaked in double distilled water for 2 h, surface sterilized with 0.1% (w/v) HgCl_2_ solution for 7 min and then washed five to seven times with sterilized water before being sown on 1/2 MS solid culture plate. After 2 weeks, the seedlings were transferred to the soil for cultivation. All *Arabidopsis* plants were grown at 22°C with long-day conditions (16-h light/8-h dark).

### Statistics of Seed Characteristics

The average weight of mature seeds was measured by using an electronic analytical balance (Sartorius, Germany). Seeds were dried under room temperature for 2 weeks. Seed weight was measured on the basis of per plant. The seed embryos were treated as previously described (Meng et al., 2017). The treated embryos were observed, and its images were captured under Carl Zeiss Microscopy GmbH (Germany). Average cotyledon (cell) area and seed size were measured by using ImageJ software. Seeds, embryos, and cotyledon cells were randomly selected and photographed under the same conditions. Two hundred seeds, cotyledons or cotyledon cells were tested in the experiments.

### Enolase Activity Assay

Enolase activity of rosette leaves was tested by using ultraviolet and visible spectrophotometer as described previously with slight modification (Straeten et al., 1991 and Eremina et al., 2015). Reactions were performed in Tris-HCl (50 mM, pH = 7.4) including 1 mM MgCl_2_ and 2.5 mM 2-phospho-d-glycerate. 1 mM PEP standard solution was used to determine the molar absorbance. Then the reaction liquids were incubated at 25°C for 10 minutes, and add 0.1 M HCl to terminate the reaction. The absorbance of the sample at 230 nm was measured. The experiments included three independent biological replicates.

### Measurement of Starch, Glucose, Sucrose, and Fructose Contents

The levels of starch, glucose, sucrose, and fructose in 0.1 g mature and dry seeds were measured on a multiskan micro-plate reader (SpectraMax Plus 384) using the starch/glucose assay kits (Solarbio^®^ Life Sciences) and sucrose/fructose assay kits (Nanjing Jiancheng Bioengineering Institute). Three independent biological and three technical replicates for each experiments were employed. Plants of each biological replicate were planted in three independent small pot, and each small pot contained two plants.

### Measurement of Phytohormones

The contents of phytohormones in 1* g* mature seeds were tested by the Shanghai Lu-Ming Biotech Co., Ltd. (Shanghai, China). In this experiment, UPLC-ESI-MS/MS was used to carry out qualitative and quantitative detection of phytohormones. Three independent biological replicates were employed.

### RNA Extraction and RNA Sequencing (RNA-Seq)

Total RNA was extracted from the 7-week-old plants (remove root), RNA concentration was quantified using Qubit^®^ 3.0 Flurometer (Life Technologies, CA, USA) and RNA integrity was assessed using RNA Nano 6000 Assay Kit on the Agilent Bioanalyzer 2100 system (Agilent Technologies, CA, USA). The cDNA library construction and sequencing were performed by Annoroad Genomics (Beijing, China) on the Illumina Hiseq Xten platform (Illumina, San Diego, CA, USA). The mRNA of two 7-week-old plants (remove root) from WT or *eno2*^−^ were sequenced as a biological relicate and three independent biological replicates were employed. The RNA-seq reads were aligned to *Arabidopsis thaliana* reference genome (TAIR 10.37) using HISAT2 (Sirén et al., 2014) and mapping rate were over 95%. Gene expression levels were assessed using Fragments per Kilobase per Million Mapped Fragments (FPKM).

### GO Annotation and KEGG Pathway Enrichment

Differentially expressed genes (DEGs) were identified by DESeq2 and the screening criteria was FC (Fold change) ≥2 and q ≤ 0.05. Genes function were annotated based on the Gene ontology (GO, http://www.geneontology.org/) and Kyoto Encyclopedia of Genes and Genomes (KEGG, https://www.genome.jp/kegg/) databases. The heatmap was drawn by using TBtools (Chen et al., 2020).

### Quantitative RT-PCR Analysis

The transcription levels of selected genes were detected by quantitative RT-PCR (real-time reverse transcription PCR) and its potential use in clinical diagnosis. Total RNA was isolated from whole plant and silique by using Eastep^®^ Super Total RNA Extraction Kit (Promega, USA), and then was reversely transcribed 1 μg RNA by using a GoScript™ Reverse Transcription system (Promega, USA). qRT-PCR was performed using TransStart Top Green qPCR SuperMix (TransGen Biotech, Chinese). Reactions were performed at 94°C for 30 s, 40 cycles of 94°C 5 s, 60°C for 30 s. qRT-PCR experiments included three independent biological replicates, each with three technical replicates. *AtUBQ5* (*Arabidopsis UBIQUITIN 5*) was used as the reference gene. The primer sequences were listed in **Table S2** of the supplementary dataset.

### Yeast Two-Hybrid Assays

The coding sequences (CDS) of ENO2 and MBP-1 of *Arabidopsis* were amplified by PCR as described previously (Kang et al., 2013). The CDS of bZIP75 (Basic-region leucine zipper 75) was cloned into pGADT7 and ENO2/MBP-1 was cloned into pGBKT7. The primers used for cloning are listed in **Table S1**. The sets of constructs were subsequently co-transformed into the yeast strain AH109. The co-transformed colonies were selected on the synthetic dextrose medium -Trp/-Leu, -Trp/-Leu/-His, and -Trp/-Leu/-His/-Ade, respectively. The AD/BD, bZIP75-AD/BD, AD/ENO2-BD, and AD/MBP-1-BD combinations were employed as negative controls.

### Expression of Recombinant Proteins and Pull Down Assay

The CDS of bZIP75 was amplified and ligated into the pGEX-6p-1 vector for the preparation of recombinant GST-bZIP75 protein. The coding sequences of ENO2 and MBP-1 were cloned into pET28a vector for the preparation of recombinant His-ENO2 and His-MBP-1 proteins, respectively. The primers used to construct recombinant proteins were listed in **Table S1**. The constructs were transformed into Escherichia coli strain BL21 (DE3). Expression was induced with 1 mM IPTG (isopropyl b-d-1-thiogalactopyranoside) at 16°C for 12 to 14 h, the recombinant proteins were purified following to the manufacturer’s protocol (Beyotime Biotechnology). According to the operating instructions of BeyoGold™ GST-tag Purification Resin (Beyotime Biotechnology), GST pull-down protein binding buffer, BeyoGold™ GST-tag Purification Resin, His-ENO2/His-MBP-1, and GST-bZIP75 (bait protein) or GST protein (negative control) were mixed and incubated at 4°C for overnight. The mixture was washed with PBS buffer for five times. The proteins were then separated by SDS-PAGE and detected by anti-His antibody.

### Luciferase Complementation Imaging Assays

Luciferase complementation imaging (LUC) assays were carried out in *N. benthamiana* leaves as described by Xu et al. (2018). The pairs of *Agrobacterium tumefaciens* strain GV3101 harboring ENO2-nLuc/cLuc-bZIP75, MBP-1-nLuc/cLuc-bZIP75, ENO2-nLuc/cLuc, MBP-1-nLuc/cLuc, nLuc/cLuc-bZIP75, and nLuc/cLuc were transformed into *Nicotiana benthamiana* L. leaves and expressed for 48 h, and the signals were detected by CCD. The list of primer sets used for the generation of constructs is included in **Table S1**.

### Bimolecular Fluorescence Complementation Assays

The coding sequences of ENO2 and MBP-1 were cloned to pSPYNE, while that of bZIP75 was cloned to pSPYCE. The primers for these constructs were listed in **Table S1**. The plasmids were transformed into *Agrobacterium tumefaciens*, which was then infiltrated into *N. benthamiana* leaves. Leaves were observed two days after infiltration under a confocal microscope (Zeiss LSM 880).

## Results

### Loss of *AtENO2* Function Reduces Seed Size

**Figure 1:**
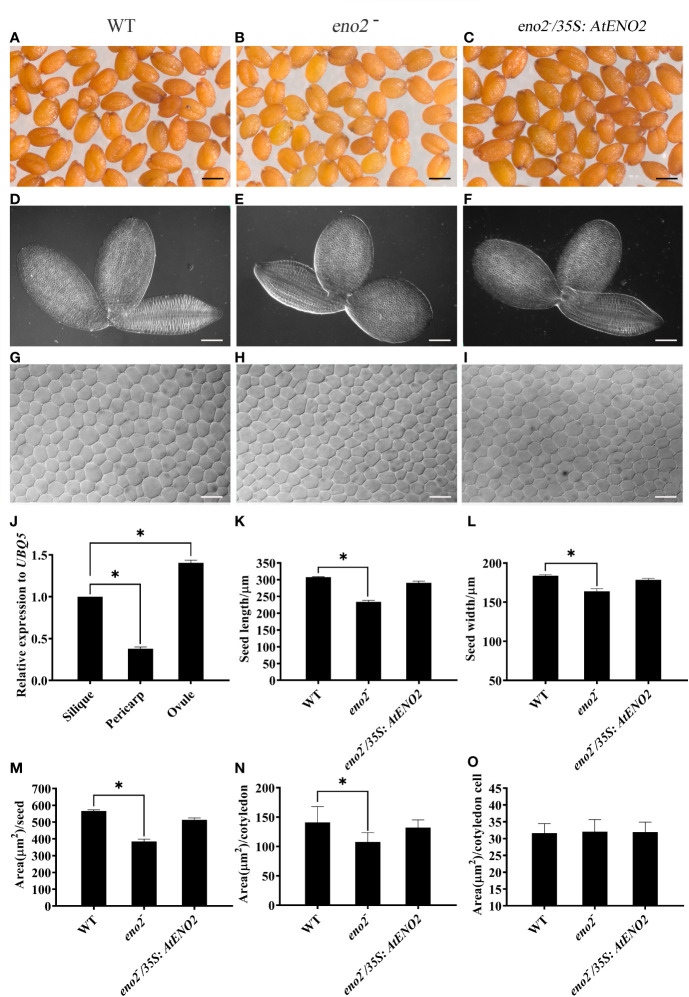
*AtENO2* regulates the seed size. The morphology of **(A–C)** seeds, **(D–F)** embryos, and **(G–I)** cells of cotyledon embryos. Seeds, embryos, and cells of cotyledon embryos were randomly selected and photographed under the same conditions. **(J)** the relative transcription levels of *AtENO2* in the silique, pericarp, and ovule of WT. The expression levels are relative to silique, which is set to 1. Error bars indicate SD values of three independent experiments. **(K, L)** Bar graph exhibiting the difference of seed length and width, respectively. **(M–O)** quantitative comparisons of seed area, cotyledon area, and cotyledon cell area among WT, *eno2*^−^, and *eno2*^−^*/35S:AtENO2*, respectively. Every group has 200 seeds. The asterisk (*) indicates the significant difference compared to WT (*P* < 0.05).

It has been reported that the expression level of *AtENO2* is much higher in roots, silique, and seeds than that in other organs which results in short silique and defective male gametophyte phenotypes in *Arabidopsis* (Andriotis et al., 2010; Eremina et al., 2015). To further check the expression pattern of *AtENO2* in the silique, we did qRT-PCR assays. We found that the expression level of *AtENO2* in ovule is significantly higher than that in pericarp (**Figure 1J**). Then we observed the characteristic of seeds in the optical microscope. As shown in **Figures 1A–C**, the seeds of WT, and *eno2*^−^*/35S:AtENO2* displayed long elliptical shape. However, e*no2*^−^ seed appeared more round in phenotype. The statistical analysis showed that the seed length, width, and area in *eno2*^−^ plants were smaller than those in WT and the expression of a 35S promoter-driven *AtENO2* construct into the *eno2* mutant (e*no2*^−^*/35S:AtENO2*) complemented the seed length, width, and size in *eno2*^−^ plants (**Figures 1K–M**). *eno2*^−^ lines also showed smaller embryos and cotyledon area than those in WT (**Figures 1D, E, N**). However, we found that cotyledon cell area of e*no2*^−^ did not show difference from that of WT (**Figures 1G–I, O**). Thus, these results indicated that the *AtENO2* plays important roles in regulation of seed size in *Arabidopsis*.

### Loss of *AtENO2* Function Reduces Seed Weight and Affects Primary Metabolism

**Figure 2:**
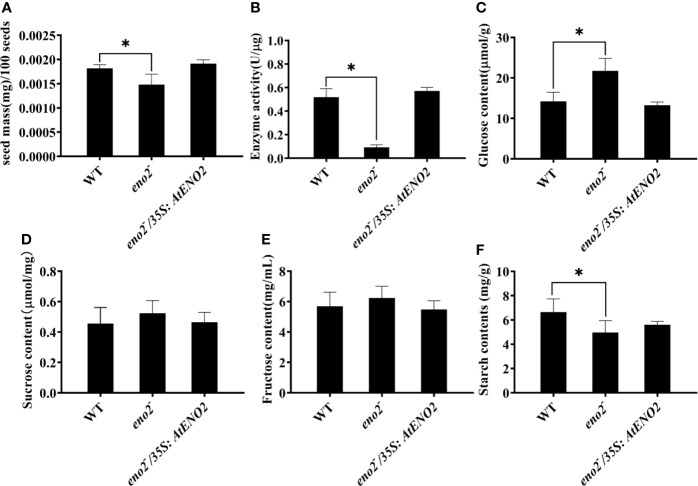
*AtENO2* affects **(A)** the seed weight, **(B)** enolase activity and contents of secondary metabolite [**(C)** glucose; **(D)** sucrose; **(E)** fructose; **(F)** starch]. The values of the bars are means of three biological replicates with three technical repeats in each biological experiment. The asterisk (*) indicates the significant differences compared to WT (P<0.05).

To further explore the *AtENO2* function, we tested the seed weight in WT, *eno2*^−^ and *eno2*^−^*/35S:AtENO2*. We found that the seed dry weight of *eno2*^−^ plants was significantly lower than that of WT and *eno2*^−^*/35S:AtENO2* (**Figure 2A**). The activity of enolase encoded by *ENO2/ENO1* plays critical roles in catalyzing the dehydration of 2-phospho-d-glycerate (2-PGA) to phosphoenolpyruvate (PEP), which produced ATP and regulated the carbohydrate metabolism (Vermerris and Nicholson, 2006; Eremina et al., 2015). Therefore, we investigated the activity of enolase in WT, *eno2*^−^ and *eno2*^−^*/35S:AtENO2* lines. As shown in **Figure 2B**, the enolase activity was drastically reduced in *eno2*^−^ plants compared to WT. The changes in enolase activity might contribute to altered carbohydrate metabolism. We next detected the contents of glucose, sucrose, fructose, and starch. We found that the levels of sucrose and fructose in *eno2*^−^ line were slightly elevated compared with other lines, while glucose contents of *eno2*^−^ line were significantly higher than that of WT and *eno2*^−^*/35S:AtENO2* plants. In contrast, starch content in *eno2* mutant was lower than compared to WT. We also found that there are no significant changes detected in the contents of glucose, sucrose, fructose, and starch between WT and *eno2*^−^*/35S:AtENO2* lines (**Figures 2C–F**).

### Identification of Differentially Expressed Genes (DEGs) in *eno2* Mutant

**Figure 3:**
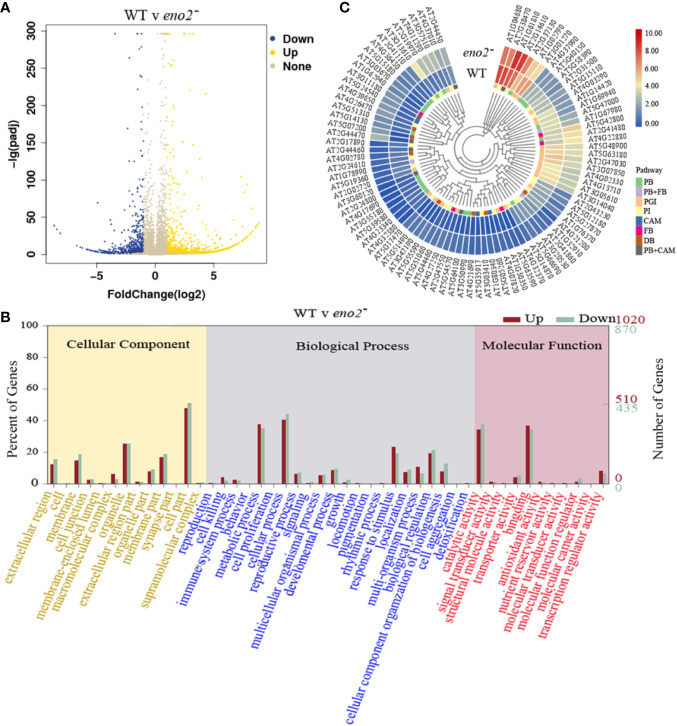
The analysis of differentially expressed genes (DEGs) in *AtENO2* mutant (*eno2*^−^). **(A)** The volcano map of DEGs. The blue and yellow dots denote significantly different expression levels in each data set (fold change > 2, P < 0.05), the gray dots indicates the no significant difference in the expression of genes. **(B)** GO term classification for DEGs. The x-axis indicates the sub-categories, the left y-axis indicates the percentage of a sub-category of genes in that category, and the right y-axis indicates the number of genes in a sub-category. **(C)** Heatmap diagrams showed the relative expression levels of DEGs among enriched KEGG pathways. PB, phenylpropanoid biosynthesis; PGI, pentose and glucuronate interconversions; PI, plant-pathogen interaction; CAM, cyanoamino acid metabolism; FB, flavonoid biosynthesis; DB, diterpenoid biosynthesis.

To investigate the molecular basis of seed development in *eno2* mutants, we employed the RNA-sequencing (RNA-seq) and obtained the DEGs by using the DESeq2 in *eno2*^−^. A total of 1892 DEGs were identified, of which 1021 and 871 genes were up-regulated and down-regulated in *eno2*^−^ plants (**Figure 3A** and **Table S2**). Among them, the most up-regulated gene was *At2g44250* (Log2FC = 8.816) which encodes a tRNA-splicing ligase. Klepikova et al. (2016) reported that it is highly expressed in *Arabidopsis* seeds. As the only known embryonic regulators required for normal development during both the morphogenesis and maturation phases, *LEC2* and *FUS3* were also up-regulated in *eno2*^−^ (**Table S2**). Alternatively, as is required for cell expansion and fruit developmental processes (Silvia et al., 2013), *At5g39280* (*AtEXPA23*, Log2FC = 8.623) and *At5g39300* (*AtEXPA25*, Log2FC = 8.384) were most down-regulated in *eno2*^−^ which may explain why AtENO2 affected the seed size.

### Gene Ontology (GO) Analysis of Differentially Expressed Genes in *eno2* Mutant

To understand the function of DEGs, we performed GO annotations analysis. The results showed that there were 56 GO terms including 22 biological process, 13 cellular component, and 21 molecular function with FDR<0.05 (**Figure 3B**). Of them, the top 2 terms were “cellular process” (411 for up-regulated DEGs, 385 for down-regulated DEGs) and “metabolic process” (383 for up-regulated DEGs, 305 for down-regulated DEGs) in the terms of biological process, respectively (**Table S3**). The GO term with the highest DEGs count number in molecular function was “cell part” (489 for up-regulated DEGs, 444 for down-regulated DEGs), followed by “cellular process” (411 for up-regulated DEGs, 385 for down-regulated DEGs) (**Table S3**).

In addition, enolase encoded by *AtENO2* plays a key role in glycolysis pathway which plays a vital role in plant metabolism. Metabolism is of considerable importance in the process of seed development. Therefore, we further checked some functional classifications of DEGs which are involved in metabolism. As shown in the **Table S4**, six representative GO categories contained 239 DEGs (146 for up-regulated DEGs, 93 for down-regulated DEGs) were identified. 31.38% of 239 DEGs (75) were related to lipid metabolism and 28.45% of 239 DEGs (68) were assigned into protein metabolism (**Table 1**). Only 7.53% of 239 DEGs (18) were enriched to amino acid metabolism (**Table 1**). These metabolic categories annotated by these well-characterized DEGs provided a valuable framework that directly links *AtENO2* to the size and weight of *Arabidopsis* seeds.

**Table 1:** Functional classification of DEGs in material metabolism.

Category	Up-regulated DEGs			Down-regulated DEGs			Total	Percentage* (%)
	Log2 (Fold Change)		Total	Log2 (Fold Change)		Total		
	≥ 2	1–2		≥ 2	1–2			
Flavonoid metabolism	11	4	15	3	3	6	21	8.79
Lipid metabolism	26	15	41	16	18	34	75	31.38
Carbohydrate metabolism	12	7	19	6	13	19	38	15.90
Fatty acid metabolism	6	8	14	3	5	8	22	9.21
Amino acid metabolism	4	8	12	3	3	6	18	7.5
Protein metabolism	41	7	48	11	9	20	68	28.45

*Percentage (%) = DEGs involved in metabolic categories/all DEGs identified in 6 representative GO category.

### KEGG Pathways of Differentially Expressed Genes

To further explore the biological functions of the DEGs, an enrichment analysis based on KEGG database was performed. The KEGG analysis revealed that 99 pathways were mapped (**Table S5**) and there were six pathways significantly enriched with 91 DGEs (P < 0.05, **Figure 3C**), including phenylpropanoid biosynthesis with 31 DEGs, pentose, and glucuronate interconversions with 23 DEGs, plant-pathogen interaction with 22 DGEs, cyanoamino acid metabolism with 10 DEGs, flavonoid biosynthesis with 8 DEGs and diterpenoid biosynthesis with 7 DEGs (**Table 2**). There were 7 DEGs assigned to phenylpropanoid biosynthesis pathway and cyanoamino acid metabolism pathway and 3 DEGs identified into phenylpropanoid biosynthesis pathway and flavonoid biosynthesis (**Figure 3C**). In these significantly enriched pathways, the most up-regulated gene is *AT2G44470* (*AtBGLU29*) which encodes a beta glucosidase and belongs to NAC transcription factors, as well as being involved in phenylpropanoid biosynthesis pathway and cyanoamino acid metabolism pathway. In addition, the most down-regulated DEG was *AT4G32380* which encodes a pectin lyase superfamily protein and participates in the regulation of pentose and glucuronate interconversions (**Table S6** of the supplementary dataset). The KEGG analysis provided a better functional insight into the DEGs and validated the GO enrichment analysis in which DEGs were predominantly correlated to seed development.

**Table 2:** Enriched KEGG pathways for DEGs in WT and *eno2*^−^ plants.

Pathway	Up-DEGs	Down-DEGs	Total DEGs	P-value	Q-value
Phenylpropanoid biosynthesis	16	15	31	4.9355E-08	3.3315E-06
Pentose and glucuronate interconversions	6	17	23	2.1603E-10	2.9165E-08
Plant-pathogen interaction	10	12	22	0.00128439	0.02477045
Cyanoamino acid metabolism	8	2	10	0.00101420	0.02281970
Flavonoid biosynthesis	5	3	8	0.00018859	0.006364979
Diterpenoid biosynthesis	5	2	7	0.00057899	0.01563272

### Expression Profiling of Transcription Factors in DEGs

Molecular studies of *Arabidopsis* and other plants have uncovered that transcription factors (TFs) play critical regulatory roles in programming seed development (Gao et al., 2016; Duan et al., 2017; Liu et al., 2017; Lian et al., 2018). To clarify the role of TFs affected by *AtENO2* in seed development, we counted the families and numbers of transcription factors differently expressed in WT and *eno2*^−^. In this work, we identified 801 TF genes belonging to 49 families in 1892 DEGs based on Plant and Transcription Factor Database (PlantTEDB, http://planttfdb.cbi.pku.edu.cn/) (**Table 3** and **Table S7**). The 490 of TF genes were up-regulated and 311 were down-regulated. What’s more, bHLH (68 DEGs), NAC (63 DEGs), and B3 (60 DEGs) families were the most abundant TF families, followed by WRKY (48 DEGs), MYB-related (46 DEGs) and ERF (46 DEGs) (**Table 3**). To date, a number of studies have examined that these TFs families induce or inhibit the expression of genes involved in plants hormone response, biotic or abiotic stresses, and vegetative growth and reproductive development of plants (Bailey et al., 2003; Olsen et al., 2005; Zhang, 2009). Based on the PlantTFDB and Le’s finding (2010) that there are 289 seed-specific genes including 48 that encodes TFs, we obtained 22 differently expression genes encoding TFs in the research work, such as *LEC2*, *FUS3*, *bZIP15* and *MYB118* (**Table 3**). Among them, ARR-B TFs family contained the most seed-specific TFs, ARR13, ARR19, ARR21, and ARR22. This family is commonly implicated as DNA-binding transcription factors in the phosphorelay-mediated cytokinin signal transduction network in higher plants (Brenner et al., 2005; Kiba et al., 2005). These results indicated that it is possible that *AtENO2* regulates the seed development by mediating the expression of transcription factors because MBP-1 protein alternatively translated from *AtENO2* has also transcriptional regulatory function.

**Table 3:** TF families of DEGs in WT and *eno2*^−^ plants.

TF family	Gene number			Seed-specific TFs
	Up-DEGs	Down-DEGs	Total	
bHLH	32	36	68	
B3	39	21	60	LEC2, FUS3
MYB-related	25	21	46	
FAR1	13	24	37	
MYB	26	10	36	MYB118, At5g23650
GRAS	12	18	30	
C3H	15	13	28	
HSF	13	7	20	At4g18870
TALE	11	5	16	
BES1	10	4	14	
NF-YB	9	4	13	NFYB9, NFYB6
Trihelix	5	6	11	
S1FA-like	2	9	11	
ARF	3	7	10	
LBD	4	5	9	
Nin-like	5	3	8	
NF-YC	5	3	8	
BBR-BPC	3	3	6	
ARR-B	3	2	5	ARR21, ARR13, ARR22, ARR19
YABBY	2	2	4	
DBB	2	1	3	
CPP	0	2	2	
Co-like	0	2	2	
NF-X1	1	0	1	
WOX	1	0	1	
NAC	39	24	63	At3g56520
WRKY	26	22	48	
ERF	25	21	46	OLEO1
C2H2	19	18	37	
bZIP	17	17	34	bZIP15, At5g07160
M-type	22	7	29	AGL91, AGL3, At3g05860
G2-like	12	9	21	
HD-ZIP	6	10	16	HDG3, HDG8, At5g07260
GeBP	13	1	14	
HB-other	11	2	13	
NF-YA	11	1	12	
Dof	6	5	11	
E2F/DP	8	2	10	
SBP	6	3	9	
GATA	5	3	8	
TCP	5	3	8	At5g41450
MIKC	3	4	7	
AP2	4	1	5	
STAT	3	2	5	
SRS	1	2	3	
LSD	2	1	3	
RAV	0	2	2	
EIL	1	0	1	
CAMTA	0	1	1	

### qRT-PCR Validation

**Figure 4:**
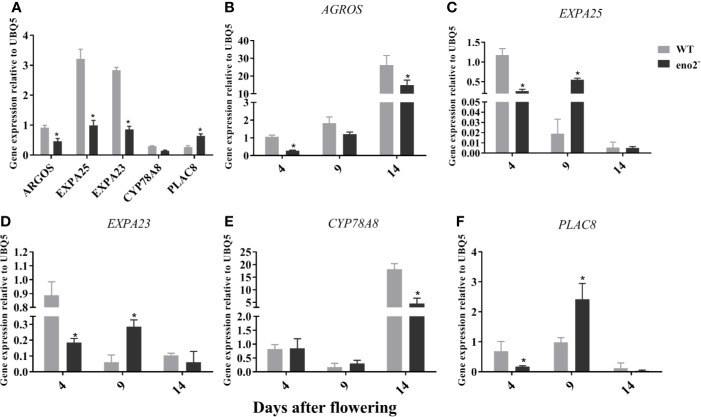
Relative expression levels of the 6 DGEs related to seed development in WT and *eno2*^−^. **(A)**: Relative expression levels of the 6 DGEs in 7-week-old *Arabidopsis* plants. **(B–F)**: Relative expression levels of each DGE in the developing silique. RNA samples were isolated from 7-week-old *Arabidopsis* plants or developing silique at three different development stages, including 4, 9, 14-days after flowering. Results were estimated by qRT-PCR using *UBQ5* as the internal standard. The gene expression levels of WT silique at 4 days after flowering were set to 1. Asterisks (*) indicates statistical significance relative to WT (P < 0.05). Three independent biological and three technical replicates were employed.

To verify the RNA-seq data, the expression profiles of 5 selected DEGs from RNA-seq assay were confirmed by qRT-PCR. As shown in **Figure 4A**, the expression of *ARGOS* (*AUXIN REGULATED GENE INVOLVED IN ORGAN SIZE*), *EXPA23* (*EXPANSIN A23*), and *EXPA25* (*EXPANSIN A25*) in 7-week-old *eno2*^−^ plants were significantly lower compared to 7-week-old WT plants and the *CYP78A8* (*CYTOCHROME P450, FAMILY 78, SUBFAMILY A, POLYPEPTIDE 8*) levels of *eno2*^−^ were slightly lower than that of WT. In contrast, the relative expression of *PLAC8* in *eno2*^−^ was significantly higher relative to the expression levels in WT. At the same time, we examined the expression patterns of these genes in the developing silique. The results displayed that the expression of *ARGOS* (*AUXIN REGULATED GENE INVOLVED IN ORGAN SIZE*) and *CYP78A8* (*CYTOCHROME P450, FAMILY 78, SUBFAMILY A, POLYPEPTIDE 8*) were highly expressed and reached a significant difference between WT and *eno2*^−^ at 14 DAF stage (**Figures 4B, E**). Meanwhile, the expression levels of *EXPA23* (*EXPANSIN A23*) and *EXPA25* (*EXPANSIN A25*) was the highest at the 4 DAF stage of WT silique but the highest at the 9 DAF stage as well as *PLAC8* (*PLACENTA-SPECIFIC 8*) (**Figures 4C, D, F**). Similarly, the transcription levels of *EXPA23* and *EXPA25* at 4 DAF were significantly higher in WT than those in *eno2*^−^ but that of these genes at 9 DAF in WT were significantly lower compared to *eno2*^−^ (**Figures 4C, D**). Moreover, the relative expression of *PLAC8* in WT was significantly lower relative to the expression levels in *eno2*^−^ at 9 DAF stage (**Figure 4F**). The results we have obtained collectively suggested that the RNA-seq data were reliable.

### Loss of *AtENO2* Function Changes the Levels of Phytohormones

**Figure 5:**
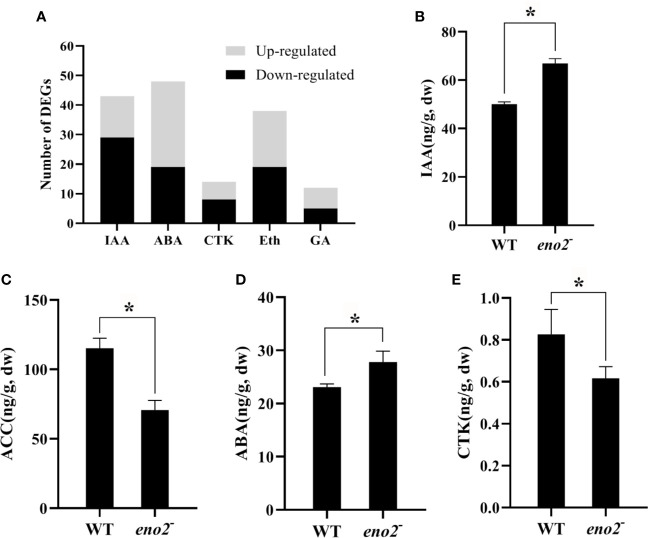
the number of DEGs related to plant hormone response pathways **(A)** and the content of phytohormones **(B–E)**. IAA, auxin; ACC, acetyl-coa carboxylase, a precursor of ethylene; ABA, abscisic acid, acid; CTK, cytokinin. The asterisk (*) indicates the significant differences compared to WT (P < 0.05).

A large number of researches have demonstrated that phytohormones directly or indirectly collaborate in plant growth and developmental processes (Cucinotta et al., 2016; Dubois et al., 2018; Miguel et al., 2020). In the RNA-seq data, we also identified 128 DEGs related to signaling pathways of five major phytohormones (**Table S7**). The response pathway of abscisic acid (ABA) contained the most DEGs (48), and the number of DEGs involved in the gibberellin-activated and biosynthesis pathways was the least (**Figure 5A**). Thus, we tested the content of five phytohormones in seeds of WT and *eno2*^−^. As shown in **Figure 5**, the levels of ABA (abscisic acid) and IAA (auxin) in the *eno2*^−^ seeds were significantly elevated than those in the WT seeds, but the content of acetyl-coa carboxylase (ACC, a precursor of ethylene) and cytokinin (CTK) were significant reduced than those in the WT seeds. The content of gibberellin was not detected. These results indicated that phytohormones push forward an immense influence on the function of *ENO2* on the seed development.

### ENO2 Interacts With bZIP75 but Not MBP-1

**Figure 6:**
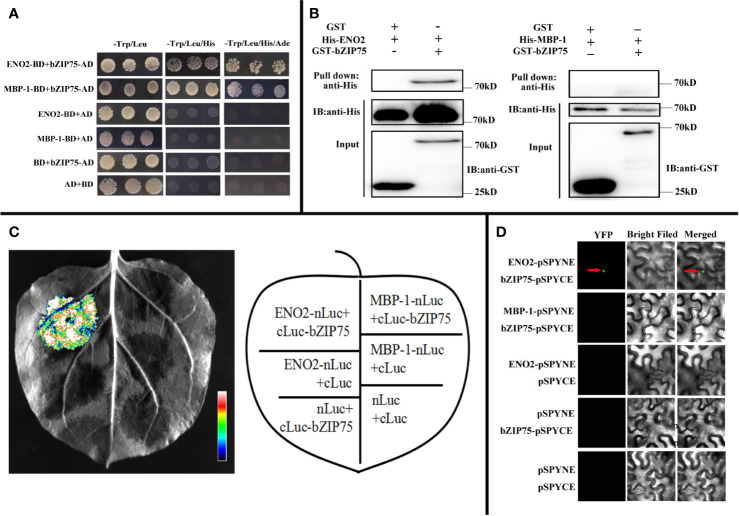
ENO2 not MBP-1 interacts with bZIP75. **(A)** Yeast two-hybrid assay demonstrating the interaction between ENO2 and bZIP75 between MBP-1 and bZIP75. ENO2-BD, MBP-1-BD, and bZIP75-AD constructs were co-transformed into Golden Yeast cells in pair-wise combinations. The AD/BD, bZIP75-AD/BD, AD/ENO2-BD, and AD/MBP-1-BD combinations were employed as negative controls. The co-transformed colonies were selected on the synthetic dextrose medium -Trp/-Leu, -Trp/-Leu/-His, and -Trp/-Leu/-His/-Ade, respectively. **(B)** Pull-down assay showing the interaction of bZIP75 and ENO2 instead of MBP-1. Purified GST-bZIP75 or GST proteins were immunoprecipitated with GST beads. Immunoprecipitated proteins were incubated with His-ENO2 or His-MBP-1. The interactions were detected by immunoblotting with an anti-His antibody. **(C)** Luciferase complementation imaging (LUC) assays uncovering only ENO2 interacts with bZIP75. ENO2-nLuc/cLuc-bZIP75, MBP-1-nLuc/cLuc-bZIP75, ENO2-nLuc/cLuc, nLuc/cLuc, nLuc/cLuc-bZIP75, and nLuc/cLuc were co-transformed into *N. benthamiana* leaves and tested after 48 h. The pseudo-color bar represents the range of luminescence intensity in the image. **(D)** Bimolecular fluorescence complementation (BiFC) assays revealing the interaction of bZIP75 and ENO2 instead of MBP-1. The construct combinations were co-transformed in *N. benthamiana* leaves and expressed for 48 h. The interaction signal was detected by confocal microscopy under 63× eyepiece.

In order to decipher the possible molecular mechanism by which *ENO2* regulates the morphology of seeds in the *Arabidopsis*, we identified the interacting proteins with ENO2 and MBP-1. We first generated the sequence that only expresses the ENO2 protein and the sequence that only expresses the MBP-1 protein according to Kang et al. (2013). Then, we used the ENO2/MBP-1 as the bait vector (ENO2-BD, MBP-1-BD) to screen the interacting proteins by performing the yeast two-hybrid (Y2H) assays. As shown in **Figure 6A**, yeast grew in the selection medium only when ENO2-BD/MBP-1-BD and bZIP75-AD were co-expressed, indicating that both ENO2 and MBP-1 may interacts with bZIP75. In order to confirm the ENO2/MBP-1-bZIP75 interaction *in vitro*, we carried out pull-down assay with GST-bZIP75 and His-ENO2/His-MBP-1-BD proteins expressed in the E. coil. The result showed that His-ENO2 could be specifically pulled down and detected with an anti-His antibody whereas the His-MBP-1 could not (**Figure 6B**). To further verify the interaction ENO2 with bZIP75 specifically, we next performed split luciferase complementation assays. *Nicotiana benthamiana* leaves cotransformed with ENO2-nLUC and cLUC-bZIP75 constructs can be detected the luciferase activity, whereas the negative control and/or MBP-1-nLUC + cLUC-bZIP75 did not have luciferase activity, indicating that ENO2 associates with bZIP75 *in vivo* (**Figure 6C**). Additional evidence that ENO2 interacts with bZIP75 came from bimolecular fluorescence complementation (BiFC) assays. The green fluorescence signal was observed in the nuclei of tobacco epidermal cells which were co-transformed into bZIP75-cYFP and ENO2-nYFP instead of MBP-1 (**Figure 6D**). These results collectively indicate that ENO2 interacts with bZIP75 in the nuclei, but not MBP-1. Compared with WT, the level of *bZIP75* expression in the *eno2*^−^ was also significantly reduced (Log2FC = −4.13) (**Table S2**).

## Discussion

*Arabidopsis ENO2* gene encodes two proteins enolase (AtENO2) and c-Myc binding protein 1-like (AtMBP-1). As a glycolytic metalloenzyme, ENO2 catalyzes the dehydration of 2-phospho-d-glycerate (2-PGA) to phosphoenolpyruvate (PEP). AtMBP-1 is a transcription factor (Liu et al., 2018). Previous researches have pointed out that the loss of *AtENO2* results in reduced growth of shoots and roots, altered vascular development, impaired floral organogenesis, and shorter silique (Eremina et al., 2015). Our results showed that the expression levels of *AtENO2* in ovule, which forms the seeds, is significantly high than that in pericarp (**Figure 2A**). As well as smaller embryo, the seeds length, width, and area of *eno2*^−^ were also smaller than those of WT (**Figures 1B, E–G**). At the same time, the seed weight of *eno2*^−^ was lighter than that of WT (**Figure 2A**). Therefore, the deficiency of *AtENO2* reduces the seed size and weight.

A possible explanation for this might be that the changes in sugar metabolism caused by *AtENO2* have a key impact on seed development. In plant cells, sugars are primarily produced from photosynthesis that functions in building large organic compounds and energy storage to facilitate chemical reactions (Sami et al., 2018). Prior studies have revealed that soluble sugar accumulation can influence seed size *via AN3-YDA* gene cascade which mediates the contents of sucrose and glucose (Meng et al., 2017). The data we obtained also displayed that *AtENO2* mutation significantly elevated the concentration of glucose and reduced the starch levels in the seed (**Figure 2**). The data available in the literature proved that the mutant of *AtENO2* elevated the levels of fructose, glucose and sucrose in the *Arabidopsis* leaves (Eremina et al., 2015). The RNA-seq analysis revealed that five DEGs were up-regulated and three DEGs were down-regulated which were involved in the conversion from cellodextrin to glucose (**Table S2**). Additionally, glycolysis products provide the substrate for the phenylpropane biosynthesis pathway. The KEGG analysis revealed that as one of the six significantly enriched pathways (**Table 2**), the phenylpropanoid biosynthesis pathway contains the most DEGs (32 DEGs) which generates an enormous array of secondary metabolites based on the few intermediates of the shikimate pathway as the core unit, such as lignin, flavonoids, and anthocyanins (Vogt, 2010). These compounds fulfill important functions, being involved in development and interaction of the plant with its environment (Petersen et al., 2010). Several reports have shown that flavonoids have been recognized as potential signal molecules regulating auxin transportation (Jacobs and Rubery, 1988) and the mutants defective in flavonoid biosynthesis pathway showed less seeds compared with wild-type (Debeaujon et al., 2000; Doughty et al., 2014), adding explanation to our results that *AtENO2* reduced seed size.

A second possible mechanism is that *AtENO2* changes the levels of phytohormones. Multiple lines of evidence indicate that sugars functions as nutrient as well as signaling molecule in plants and the function of sugar signals is to facilitate plant growth and development by interacting with phytohormones (Leon and Sheen, 2003; Gibson, 2003; Barbier et al., 2015; Gupta et al., 2015). It is worth noting that there are 60 DEGs belonging to B3 domain transcription factors which have a pivotal role in the regulation of embryo maturation and hormone signaling pathways in the seed (Suzuki and McCarty, 2008). *LEC2* and *FUS3*, as the members of B3 domain TFs, were differentially expressed in *AtENO2* mutants (**Table S2**). They are unique in that they are the only known embryonic regulators required for normal development during both the morphogenesis and maturation phases (Harada, 2001). Therefore, we speculated that *ENO2* affects the level of plant hormones. LC-MS/MS results evinced that the mutation of *ENO2* decreased the contents of cytokinin in the seeds (**Figures 5C, E**). Cytokinin regulates cell division and differentiation and a positive crosstalk were observed between glucose and cytokinins (CKs) that modulate the growth of hypocotyl and root, embryogenesis, vascular morphogenesis, and anthocyanin production (Tomáš and Thomas, 2009; Kushwah et al., 2011; Sunita and Ashverya, 2013). In this study, *eno2*^−^ lines displayed smaller embryos and cotyledon area, but no difference in cotyledon cell area than those in WT (**Figure 1**). Our research results indicated that the number of cotyledon cells in *eno2*^−^ is less than that of cotyledon cells in WT. Therefore, we inferred that *ENO2* reduces the number of cells by decreasing the content of cytokinin, thereby reducing the seed size. RNA-seq analysis displayed that ARR-B TFs families contained the most TFs (ARR13, ARR19, ARR21, ARR22) in 22 DEGs encoding seed-specific TFs in our work (**Table 3**). Argueso et al. (2010) pointed out that type-B response regulators (ARR) act as major players in the transcriptional activation of cytokinin-responsive genes. This also reveals that cytokinin plays an important role in seed size though regulating by *ENO2*.

Cytokinins play many functions in plant development, often acting in concert with other hormones, most notably auxin, to regulate cell division, and differentiation (Kieber and Schaller, 2018). In our work, the absence of *ENO2* increased the levels of abscisic acid and auxin but decreased the contents of ethylene in the seeds (**Figures 5B, D**). In the development of seeds, abscisic acid (ABA) mainly regulates the synthesis of seed storage proteins and lipids, the promotion of seed desiccation tolerance and dormancy, and the inhibition of the phase transitions from embryonic to germinate growth (Finkelstein et al., 2002). Both increased auxin and decreased ethylene content delay fruit ripening (Pech et al., 2012; Sravankumar et al., 2018). This provides an explanation for the *ENO2* mutation delaying the maturation of silique (**Figure S1**).

In addition, protein interaction experiments convinced that ENO2 interacts with bZIP75 which belongs to bZIP (**Figure 6**). Compared with WT, the level of *bZIP75* expression in the *eno2*^−^ was also significantly reduced (Log2FC = −4.13) (**Table S2**). In plant, basic region/leucine zipper motif (bZIP) transcription factors (TFs) are involved in the regulation of various biological processes from development to stress response (Liu H. J. et al., 2019). There are 75 bZIP TFs in *Arabidopsis*, which are classified into ten groups and bZIP75 belongs to S group (Jakoby et al., 2002). Although the detailed function of bZIP75 in plant growth and development is not known, several other members of S group have been reported. The literature demonstrated that bZIP53 regulated the expression of *Arabidopsis* seed maturation genes based on heterodimerization with bZIP10 or bZIP25 (Rosario et al., 2009), and regulated amino acid metabolism by interacting with bZIP1 to target the promoter of *ASPARAGINE SYNTHETASE 1* (*ANS1*) and *PROLINE DEHYDROGENASE* (*ProDH*) which are also targeted by bZIP11 (Hanson et al., 2008; Dietrich et al., 2011). Over-expression of *OsbZIP58* increased the accumulation of starch by bind directly to the promoters of six starch-synthesizing genes (Wang et al., 2013). Transgenic plants overexpressing *bZIP3* and dominant repressor form bZIP3-SRDX displayed aberrant shaped cotyledons with hyponastic bending (Sanagi et al., 2018). It is rather remarkable that the expression of these bZIPs belonging to S group was repressed by sucrose-specific signaling (Rook et al., 1998; Kang et al., 2010; Sanagi et al., 2018). Besides the results that bZIP75 interacted with ENO2 but not MBP-1 in our study, the sucrose content in *eno2*^−^ is slightly higher than that of WT (**Figure 2D**), and the expression of *bZIP75* is significantly decreased due to mutation of ENO2 (**Table S2**). Therefore, we inferred that bZIP75 may be involved in sugar signaling and *ENO2* affects the seed size and weight by forming ENO2-bZIP75 complex to regulated primary and secondary metabolism in *Arabidopsis thaliana*. Of course, the role of bZIP75 in seed development needs to be further studied.

## Conclusion

**Figure 7:**
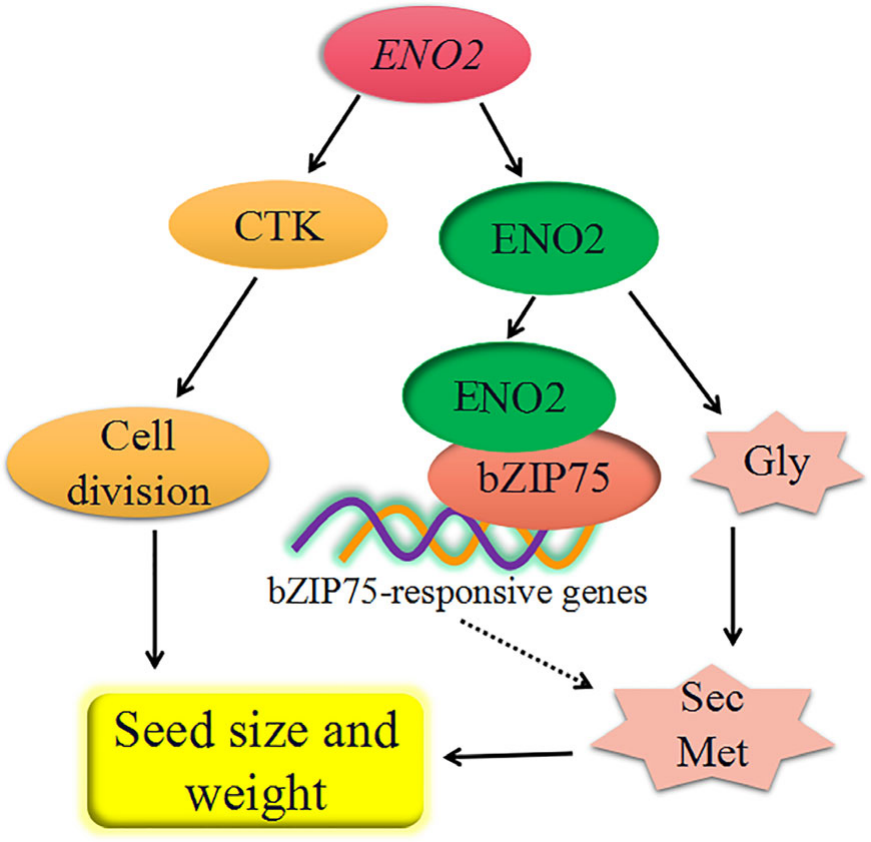
the proposed diagram of major regulatory network affected by *AtENO2* in *Arabidopsis* seed development. *ENO2* increases the accumulation of cytokinin (CTK) further to promote cell division. ENO2 translated from *ENO2* gene affects the secondary metabolism (Sec Met) by promoting glycolysis (Gly). The effects of enolase on secondary metabolism may also be regulated by the interaction of ENO2 and bZIP57. Dashed lines indicate putative signaling pathways.

Based on our results and previous studies, we constructed a model summarizing the factors responsible for the effect of *AtENO2* on the seed size and weight (**Figure 7**). The T-DNA insertion of *ENO2* reduced seed size in *Arabidopsis thaliana*, mainly affected by decreasing the content of CTK to inhibit cell division. The fact that seed size and weight of *AtENO2* mutants is smaller and lighter than that of WT is also partly explained by the defective glycolysis pathway which is regulated by ENO2 (enolase). The defective glycolysis pathway in the seeds of *AtENO2* mutants reduces the substrates to phenylpropanoid biosynthesis pathway, which further inhibits the secondary metabolism, such as lignins, anthocyanins, flavonoids, and flavonols. Moreover, the effect of *ENO2* on seed development may also be regulated by the interaction of ENO2 and bZIP75 to may mediate the secondary metabolism. Further investigations are necessary to reveal the molecular mechanism based on an ENO2-bZIP75 regulatory module for seed size and weight in *Arabidopsis thaliana*. In generally, our results demonstrated that *AtENO2* plays a crucial function in shaping the seed size and determining the seed weight.

## Data Availability Statement

The datasets (RNA-seq) for this study can be found in the National Center for Biotechnology Information (accession number: PRJNA600328).

## Author Contributions

GZ and ZL designed the experiments. ZL and LZ performed the most of experiments and analyzed the data. ZL wrote the paper with the assistance of LP. Other authors assisted in experiments and data analysis. All authors contributed to the article and approved the submitted version.

## Funding

This work was supported by the National Natural Science Foundation of China (31872672 and 31470399).

## Conflict of Interest

The authors declare that the research was conducted in the absence of any commercial or financial relationships that could be construed as a potential conflict of interest.

## Supplementary Material

Table S1 Primers used for qRT-PCR analysis.

Table S2 A list of DEGs between WT and *eno2*^−^.

Table S3 Numbers of DEGs involved in GO terms.

Table S4 A list of DEGs which were involved in 6 representative GO category related to metabolism.

Table S5 A list of KEGG pathway.

Table S6 A list of DEGs involved in significantly enriched pathways.

Table S7 A list of DEGs which were related to plant hormones.

Figure S1 Maturation rate of silique in WT and *eno2*^−^ at the different growth stages. “D” respresents days that Arabidopsis thaliana grows in the soil.
