# Supplementary figures and images for: ENO2 Affects the Seed Size and Weight by Adjusting Cytokinin Content and Forming ENO2-bZIP75 Complex in Arabidopsis thaliana
Source: Front Plant Sci. 2020 Aug 26;11:574316. doi: 10.3389/fpls.2020.574316 (PMC7479207; doi:10.3389/fpls.2020.574316)

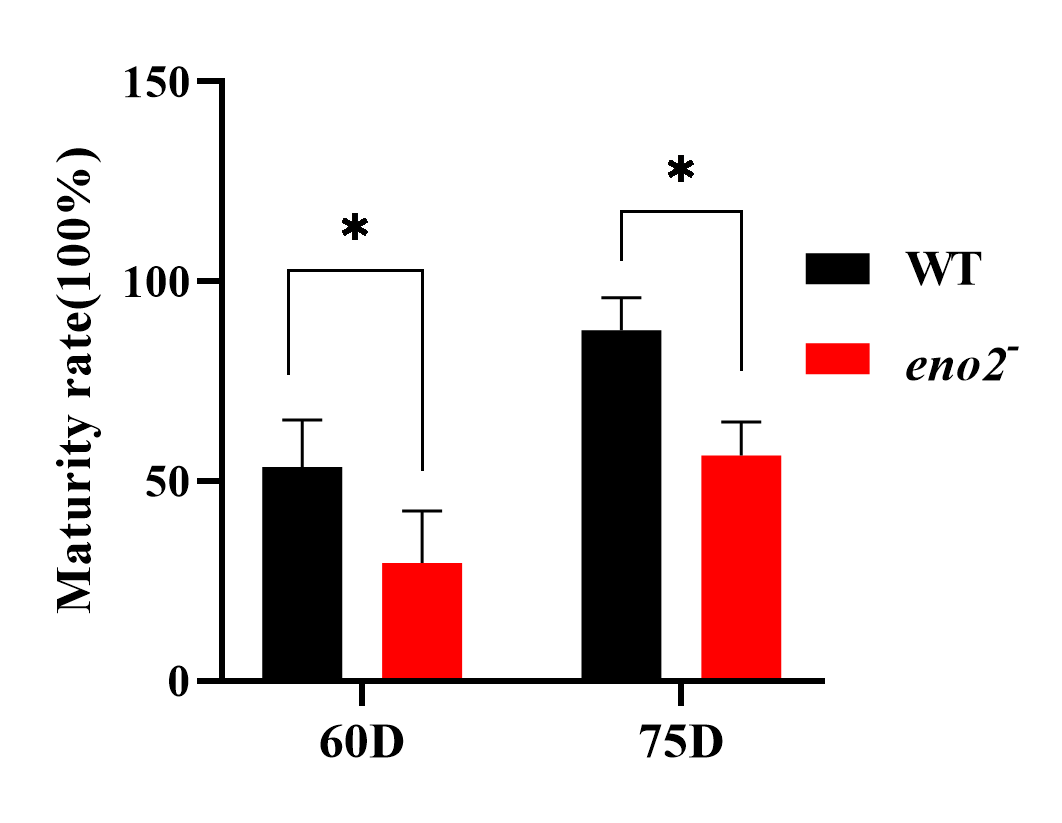

Supplement: Figure S1 — Maturation rate of silique in WT and eno2− at the different growth stages. “D” respresents days that Arabidopsis thaliana grows in the soil. [file Image_1.tif]
